# Myoglobin Protects Breast Cancer Cells Due to Its ROS and NO Scavenging Properties

**DOI:** 10.3389/fendo.2021.732190

**Published:** 2021-10-04

**Authors:** Theresa Quinting, Anna Katharina Heymann, Anne Bicker, Theresa Nauth, Andre Bernardini, Thomas Hankeln, Joachim Fandrey, Timm Schreiber

**Affiliations:** ^1^ Institute of Physiology, University of Duisburg-Essen, Essen, Germany; ^2^ Institute of Organismic and Molecular Evolution, Molecular Genetics and Genome Analysis, Johannes Gutenberg University, Mainz, Germany; ^3^ Institute of Physiology, Pathophysiology and Toxicology and Center for Biomedical Education and Research, University of Witten/Herdecke, Witten, Germany

**Keywords:** myoglobin, breast cancer, HIF, hypoxia, ROS, NO, tumor

## Abstract

Myoglobin (MB) is an oxygen-binding protein usually found in cardiac myocytes and skeletal muscle fibers. It may function as a temporary storage and transport protein for O_2_ but could also have scavenging capacity for reactive oxygen and nitrogen species. In addition, MB has recently been identified as a hallmark in luminal breast cancer and was shown to be robustly induced under hypoxia. Cellular responses to hypoxia are regulated by the transcription factor hypoxia-inducible factor (HIF). For exploring the function of MB in breast cancer, we employed the human cell line MDA-MB-468. Cells were grown in monolayer or as 3D multicellular spheroids, which mimic the *in vivo* avascular tumor architecture and physiology with a heterogeneous cell population of proliferating cells in the rim and non-cycling or necrotic cells in the core region. This central necrosis was increased after MB knockdown, indicating a role for MB in hypoxic tumor regions. In addition, MB knockdown caused higher levels of HIF-1α protein after treatment with NO, which also plays an important role in cancer cell survival. MB knockdown also led to higher reactive oxygen species (ROS) levels in the cells after treatment with H_2_O_2_. To further explore the role of MB in cell survival, we performed RNA-Seq after MB knockdown and NO treatment. 1029 differentially expressed genes (DEGs), including 45 potential HIF-1 target genes, were annotated in regulatory pathways that modulate cellular function and maintenance, cell death and survival, and carbohydrate metabolism. Of these target genes, *TMEFF1*, *TREX2*, *GLUT-1*, *MKNK-1*, and *RAB8B* were significantly altered. Consistently, a decreased expression of *GLUT-1*, *MKNK-1*, and *RAB8B* after MB knockdown was confirmed by qPCR. All three genes of interest are often up regulated in cancer and correlate with a poor clinical outcome. Thus, our data indicate that myoglobin might influence the survival of breast cancer cells, possibly due to its ROS and NO scavenging properties and could be a valuable target for cancer therapy.

## 1 Introduction

Myoglobin (MB) is a cytoplasmic, oxygen-binding heme-protein mainly found in cardiac myocytes and skeletal muscle fibers These genes are often up regulated in cancer and correlate with a poor clinical outcome where it functions as a mobile oxygen reservoir. In times of high oxygen demand, MB is able to provide mitochondria with oxygen through its reversible O_2_ storage and transport function (1, 2). Besides these long-known functions, newer evidence showed that MB is a capable regulator of nitric oxide (NO) homeostasis in muscle cells by either scavenging (3) or producing NO (4, 5). Other attributed functions of MB now include scavenging of reactive oxygen species (ROS) (6), and binding and transport of fatty acids and acylcarnitines (7, 8). To date it is known that MB is not only strongly expressed in muscle tissue and myocytes, but also endogenously at lower levels in different forms of malignant tumors, like prostate cancer, non-small cell lung cancer, renal cell carcinoma, and breast cancer (9–12). Kristiansen et al. analyzed the appearance of MB in a large comprehensive study of invasive and non-invasive breast cancer cases and found ~40% of invasive carcinomas being at least moderately positive for *MB* expression. MB was preferentially detected in hormone receptor-positive tumors and Kaplan-Meyer analysis revealed a better prognostic outcome for patients with high MB levels (12). *MB* mRNA was furthermore detected at different levels in a subset of human breast cancer cell lines, like MDA-MB-468 (high-*MB*) or MCF-7 (low-*MB*) (12). In breast cancer cell lines, MB RNA and protein levels were inducible by prolonged hypoxia, which was in part attributed to the transcriptional activation by the hypoxia-inducible factors-1 and -2 (HIF-1, HIF-2) (13). *MB* transcription is thereby started from a hypoxia-inducible alternative upstream promoter region that is different from the myocytic standard promoter and could be silenced by estrogen hormone treatment (14, 15). Studies showed a lower cellular O_2_ uptake in breast cancer cells after MB knockdown (KD) as well as a decreased mitochondrial dehydrogenase activity, leading to the assumption that the occurrence of MB in tumor cells is not mainly linked to its main function as an O_2_ storage and transport protein (13). Regulation and function of MB could therefore be fundamentally different between muscle and tumor tissue.

One of the main activators of cellular responses to hypoxia are HIFs, which induce the transcription of target genes mainly involved in angiogenesis, erythropoiesis and glycolysis, and also play an important role for the cellular adaptation of the tumor microenvironment (16, 17). HIFs (HIF-1, HIF-2, and HIF-3) are heterodimeric transcription factors consisting of an oxygen sensitive alpha-subunit that is degraded by the proteasome under normoxic conditions, and a nuclear, unregulated beta-subunit (18). Hypoxic regions are a common feature of many solid tumors (19) with a poorer prognosis because of a higher aggressiveness and progression of the tumor, and resistance to irradiation and chemotherapy (20, 21). Here, HIF-1α serves as a marker for hypoxia correlated with malignant properties and increased patient mortality in many different types of cancer (21). In mamma carcinomas, HIF-1α-positivity is associated with higher proliferation rates and tumor size, higher grading status, shorter metastasis-free intervals, and negative hormone receptor status (22). Of note, not all breast cancer samples expressing *HIF-1α* are necessarily hypoxic. Vleugel et al. showed partially diffuse HIF-1α staining patterns in all regions of the tumor, indicating alternative activation pathways of HIF-1α (23). Indeed, there is a general link between HIF-1α stabilization and other inductors besides hypoxia. Induction of HIFs can occur through inflammatory cytokines or growth factors (24, 25) as well as interaction with the NO signaling pathway. NO is thereby only indirectly affecting HIF, but can interact with and inhibit oxygen-sensor enzymes like prolyl-hydroxylases, which normally target the HIF alpha subunit for proteasomal degradation under normoxic conditions (26). Other activation mechanisms of HIF response include the interaction with H_2_O_2_ and other ROS (27, 28).

In 2020, breast cancer was the most frequently diagnosed cancer in women worldwide with many known risk factors, and the most common cause of cancer-related death in women in 110 countries (29). To date the exact role of MB in tumors in general and breast carcinoma in particular is not fully understood. A positive correlation between MB and HIF-1α target gene expression was shown in breast, prostate, and non-small cell lung cancer (9, 10, 12, 30). By using an unbiased transcriptomics approach in combination with a siRNA-induced knockdown of MB and hypoxic versus normoxic incubation, a follow-up study reported an influence of MB on key metabolic and regulatory processes in these cancer cell lines. The RNA-Seq data suggested a functional impact of MB on ROS and NO homeostasis, correlating MB positively with increased HIF-1α and p53 signaling (31).

To test and extend the proposed working models, we investigated how MB influences HIF-1α and NO signaling pathways and cell survival by using the high-MB breast cancer cell line MDA-MB-468 in a monolayer and tumor spheroid cell culture model. When comparing wildtype (WT) cells to MB knockdown (KD) cells achieved by transient siRNA treatment, we found an increased central necrosis in tumor spheroids after MB KD, indicating that a lack of MB stimulates cell death. Furthermore, MB KD caused higher levels of HIF-1α protein after treatment with NO and higher reactive oxygen species (ROS) levels in the cells after treatment with H_2_O_2_. As HIF-1 is a transcription factor with several hundred target genes, we performed RNA-Seq analysis after MB KD and NO treatment to identify altered mRNA expression patterns. Of 45 potential HIF-1 target genes that were annotated in regulatory pathways that modulate cellular function and maintaining, cell death and survival, and carbohydrate metabolism, we identified a significant down regulation for *GLUT-1*, *MKNK-1* and *RAB8B* which was verified by qPCR. All three genes are often up regulated in cancer and correlate with a poor clinical outcome (32–34). Based on our data, myoglobin therefore might influence the survival of breast cancer cells, possibly due to its ROS and NO scavenging properties and could be a valuable target for cancer therapy.

## 2 Materials and Methods

### 2.1 Cell Culture Under Normoxia and Hypoxia

For all experiments, the human mammary epithelial cell line MDA-MB-468 (ATCC HTB-132, verified by Leibniz Institute DSMZ, Braunschweig) was used as monolayer culture or in a three-dimensional tumor spheroid model. Cells were cultured in DMEM (Gibco) supplemented with 10% FCS (Sigma) and 1% Penicillin/Streptomycin (Gibco) under standard conditions (37°C, 5% CO_2_) in water-saturated room air (21% O_2_; normoxia). Depending on the experimental set-up, hypoxic cultivation was performed for up to 72 h under water-saturated 1% O_2_ with 5% CO_2_ and balanced N_2_ at 37°C using the InvivO_2_ 400 hypoxia workstation + gas mixer Q (Baker Ruskinn).

### 2.2 Three-Dimensional Tumor Spheroids

#### 2.2.1 Generation of Spheroids

Tumor spheroids of MDA-MB-468 cells were generated as described elsewhere (35). Briefly, cells were seeded in 6-well plates at 1x10^5 cells/ml and incubated for 24 h under standard conditions. Cells were harvested and mixed with 5% ice-cold reconstituted basement membrane (rBM, MatrigelTM) before transferring 200 µl of the cell suspension/2x10^4 cells into each well of 0.5% poly-HEMA coated 96-well plates. Spheroid formation was initiated by 10 min of centrifugation (1000 rpm, 4°C) and the spheroids then incubated for 24 h until beginning of experiments.

#### 2.2.2 Central Necrosis

24 h after initial seeding, tumor spheroids were incubated for 24 h, 48 h or 72 h under normoxic or hypoxic conditions and the central necrosis of the spheroids was measured. For this, spheroids were treated for 4 h with 0.5 µg/ml 4′,6-diamidino-2-phenylindole (DAPI) for the detection of dead cells. To test the effects of different stress-inducing substances on the formation of a central necrosis, spheroids were furthermore treated with H_2_O_2_, NO or a combination of both (H_2_O_2_ + NO) 24 h after initial seeding and incubated for 4 h under normoxic conditions before DAPI staining. As one further condition, spheroids were irradiated with 5 Gy using an X-ray machine (X-rad 320, PXI) operated at 320 kV and 12.5 mA with a 1.65 mm aluminum filter to mimic irradiation therapy of tumor cells. Fluorescence was measured with a Zeiss Axiovert 200M microscope and DS-Ri1 camera system using the NIS-elements F 3.0 imaging software. DAPI signal was analyzed as pixel/field of view using ImageJ for each tumor spheroid.

### 2.3 S-Nitrosoglutathione (GNSO) Exposure

S-Nitrosoglutathione (GNSO) was synthesized as a 1 mM stock solution as previously described (36) and used as a source for bioavailable NO. For experiments, spheroids were cultivated as described or monolayer cells were seeded at 100.000 cells/ml in 6-well plates and incubated for 24 h under either normoxic or hypoxic conditions before addition of 500 µM GNSO. After 0 - 6 h of further normoxic or hypoxic incubation, respectively, samples were taken for further RNA or protein analysis.

When introducing the transient *MB* knockdown *via* RNA interference, cells were seeded for 24 h as mentioned above, followed by transfection with the respective siRNA for another 24 h under normoxic conditions before starting the GNSO treatment and sample uptake.

### 2.4 Transient RNA Interference

#### 2.4.1 MB Knockdown

Cells were transfected with Hs_MB_6 FlexiTube siRNA (Catalog No. SI04235945) for a transient knockdown of MB (=KD) or with allStars Negative Control scrambled RNA (Catalog No. 1027280; Qiagen, Hilden, Germany) as control (=WT). HiPerFect (Catalog No. 301704; Qiagen, Hilden, Germany) was used as transfection reagent orientated towards the protocol provided. Briefly, 2.3x10^5 cells were seeded on a 6-well plate and incubated for 24 h under standard conditions. For transfection, medium was changed to serum-free conditions and cells incubated with the transfection complex (100 µl serum-free medium + 10 µl siRNA + 12 µl HiPerFect transfection reagent, left at room temperature for 10 min for complex formation) for 20 min at room temperature. Next, medium was directly changed to serum-containing medium again and monolayer cells were left incubated depending on the experimental set-up. For spheroids, spheroid formation was introduced 4 h after transfection according to the protocol above. MB knockdown efficiency was validated by qPCR with efficiencies of up to 90% for both monolayer and spheroid cultures under both normoxic and hypoxic incubation conditions.

#### 2.4.2 pC1-HyPer3 Plasmid

2x10^5 cells were seeded into WillCo-dish^®^ glass bottom dishes (WillCo Wells B.V, Amsterdam, Netherlands) and first transfected with siRNA according to the protocol above. After 24 h of incubation under standard conditions, the second transfection took place using the ViaFect transfection reagent (Promega) and 2 µg of pC1-HyPer3 plasmid, a genetically modified H_2_O_2_ probe [Addgene plasmid # 42131 (37)] for detection of ROS. The transfection complex (200 µl serum-free medium + 4.3 µl plasmid DNA + 4 µl ViaFect transfection reagent) was formed for 13 min at room temperature before adding to the cells without changing of medium. After 24 h of further standard incubation, cells were prepared for ROS measurements as mentioned below.

### 2.5 ROS Measurements

For performing live cell imaging, the medium of the double-transfected cells (MB knockdown + pC1-HyPer3 plasmid) was changed to FluoroBrite™ DMEM (Gibco). Before measurement, ROS formation was introduced by addition of 100 µM H_2_O_2_ to the cells directly under the microscope (Nikon Eclipse Ti). Fluorescence was measured using a 565/40 nm and 480/30 nm filter set and the mean intensity as pixel/field of view was analyzed using ImageJ. Data are represented as quotient of 565/480.

### 2.6 RNA Extraction, Reverse Transcription and Quantitative Real-Time PCR (qPCR)

Cells were lysed using a 4 M guanidinium thiocyanate solution and RNA extracted using the RNeasy Mini Kit (Qiagen GmbH, Germany) according to the protocol provided. A total of 1 µg RNA was transcribed into cDNA using oligo-dT-nucleotides (Life Technologies, USA) as primers and M-MLV reverse transcriptase (Promega). Quantitative real-time PCR (qPCR) was performed using the MESA Green qPCR MastermixPlus for SYBR^®^ ASSAY (Eurogentec) on an iCycler iQ5 multicolor real-time PCR detection system (Bio-Rad) using the following program: initial denaturation for 10 min at 95°C, then 45 cycles of denaturation (15 sec, 95°C), and elongation (60 sec, 60°C). Gene expression was analyzed using the 2^-∆∆CT^ (cycle threshold) method with ribosomal protein S16 (rP) serving as a reference gene. Please refer to **Table 1** for respective primers.

**Table 1:** Primers for qPCR.

Primers (gene)	5’ sequence	3’ sequence
*HIF-1α*	CAT AAA GTC TGC AAC ATG GAA GGT	ATT TGA TGG GTG AGG AAT GGG TT
*Myoglobin (MB)*	AGT TGG TGC TGA ACG TCT GG	GGT GAC CCT TAA AGA GCC TGA
*Ribosomal protein S16 (rP)*	AGA TGA TCG AGC CGC GC	GCT ACC AGG GCC TTT GAG ATG GA

### 2.7 Protein Extraction and Western Blots

For protein extraction, cells were harvested with lysis buffer (0.5% NP40, 150 mM NaCl, 10 mM Tris pH 7.9, 2 mM EDTA, 10% protease inhibitor) and total protein concentration was measured with a bovine serum albumin (BSA) standard and the DC Protein Assay kit (Bio-Rad). For Western blotting, 40 µg of each protein extract was electrophoresed on a 7.5% SDS-PAGE gel before transfer to a nitrocellulose membrane. The membrane was blocked with 5% nonfat dry milk in 0,1% TBS-T for 1 h at room temperature before incubation with primary antibody at 4°C overnight. Secondary antibodies were incubated for 1 h at room temperature. Antibodies are listed in **Table 2**. For visualization, the ECL AdvanceTM Western Blotting Detection Kit (GE Healthcare) was employed. Signals were detected on a photo film. If applicable, membranes were stripped in Restore™ PLUS Stripping Buffer (Thermo Scientific). Quantification of protein signals was performed using ImageJ.

**Table 2:** Antibodies used for standard Western blotting.

	Concentration	Species
Primary antibody		
Anti-Actin (Abcam, polyclonal)	1:1000	Rabbit
Anti-HIF-1α (Cayman, polyclonal)	1:1000	Rabbit
Anti-hydroxy-HIF-1α (Pro564) (D43B5) XP^®^ (Cell signaling, monoclonal)	1:500	Rabbit
Secondary antibody		
Anti-rabbit HRP conjugate (Sigma)	1:100 000	Goat

Concentrations implicate antibody dilution in 0.1% TBS-T.

#### 2.7.1 Subcellular Fractioning

MDA-MB468 and LNCaP cells were cultured for 72 h at normoxia or hypoxia. About 5x10^7 cells were washed with PBS (PAA) supplemented with protease inhibitor and harvested from culture flasks with a cell scraper (Sarstedt). After 10 min centrifugation at 500xg at 4°C, subcellular fractionation was conducted with the Qproteome Cell Compartment kit (Qiagen). Protein samples were precipitated in four volumes of ice-cold acetone and solved in PBS. Protein concentration was determined by bicinchoninic acid assays (BCA), using the BCA Protein Assay reagent (Pierce) with BSA standard dilutions for quantification. Samples were electrophoresed on 12% SDS polyacrylamide gels and blotted to a nitrocellulose membrane. Membranes were incubated in 5% non-fat dry milk for 1 h and washed in 0.1% PBS-T for 5 min. Primary antibodies were applied (**Table 3**) overnight at 4°C. Secondary antibodies (**Table 3**) were incubated for 1 h at room temperature. ECL solution was applied for time frames varying from 45 sec to 5 min to detect light emission *via* photo film (Kodak) exposure. Membrane stripping was conducted with Mild Stripping buffer (Abcam).

**Table 3:** Antibodies used for Western blotting of subcellular fractions.

	Concentration	Species
Monoclonal primary antibody		
Anti-Actin (Sigma-Aldrich)	1:1000	Rabbit
Anti-Histone H3 (Cell Signaling)	1:1000	Rabbit
Anti-Vimentin (abcam)	1:750	Mouse
Anti-GM130 (BD Biosciences Pharmingen)	1:200	Mouse
Anti-Myoglobin (DakoCytomation)	1:500	Rabbit
Anti-COX4 (abcam)	1:250	Mouse
Anti-α-Tubulin (Sigma-Aldrich)	1:500	Rabbit
Secondary antibody		
Anti-Rabbit IgG, HRP conjugate (R-05072-500 Advansta)	1:250 000	Goat
Anti-Mouse IgG, HRP conjugate (541089 Biozym Scientific)	1:250 000	Goat

Concentrations implicate antibody dilution in 0.1% PBS-T.

### 2.8 RT^2^ Profiler PCR Array

Cells were seeded at 100.000 cells/ml in 6-well plates and incubated for 24 h under normoxic conditions before transfection with the respective siRNA for another 24 h under normoxia. For the array, cells were then treated with 500 µM GNSO for 3 h before cell lysis and RNA extraction. With the RT² Profiler™ PCR Array Human Hypoxia Signaling Pathway (Qiagen), the expression of 84 genes related to hypoxic signaling was analyzed in a 96-well plate qPCR format according to the protocol provided.

### 2.9 RNA-Seq

For RNA-Seq analyses, GNSO-treated (500µM) or untreated KD and WT cells were raised under normoxia or hypoxia (1% O_2_ for 6 h). RNA was extracted (see above), quality-checked on an Agilent RNA 6000 nano Bioanalyzer Chip and quantified with the Qubit RNA BR assay. RNA-Seq libraries were generated with the NEBNext^®^ Ultra™ II directional dual index RNA library preparation kit (StarSEQ Mainz). Single-end 75 nt reads were sequenced on an Illumina Next Seq 500 platform (StarSEQ Mainz) and demultiplexed. Raw reads were filtered for adapters and low-quality sequences (below Phred 13), allowing up to two ambiguous nucleotides per read with the CLC Genomics Workbench 11.0.1 Trimmer. Remaining reads were mapped against the human genome hg19 (ftp.ensembl. org/pub/release-75/gtf/homo_sapiens/Homo_sapiens.GRCh37.75. gtf.gz) with the RNA-Seq analysis tool of the CLC Genomics Workbench 11.0.1. Mapping parameters included intergenic regions and allowed up to 10 hits per read. Differentially expressed genes between KD and WT cells were identified for each experimental condition by paired two-group comparisons of read counts, using the CLC Genomics Workbench 11.0.1. Proportion-based statistics were calculated with Kal’s Z-tests [Bonferroni- and false discovery rate (FDR)-corrected]. Gene lists with TPMs and p-Values were interpreted by Ingenuity pathway analysis (IPA), including the IPA *a priori* knowledge of direct and indirect relationships between genes for all human tissues. RNA-Seq data are accessible at ENA (EBI). Study number is PRJEB45683.

### 2.10 Statistical Analyses

All data except for RNA-Seq analyses was analyzed using GraphPad Prism 7 software (GraphPad Software Inc., San Diego, USA, RRID : SCR_002798). All graphs are shown as mean +/- standard deviation. Statistical significance was calculated by unpaired Student’s t-test or two-way ANOVA with post-hoc Sidak’s multiple comparisons test when having two categorical factors to compare. P values < 0.05 were considered statistically significant.

## 3 Results

### 3.1 MB Is Found in the Cytoplasm of Breast Cancer Cells Under Both Normoxia and Hypoxia

**Figure 1:**
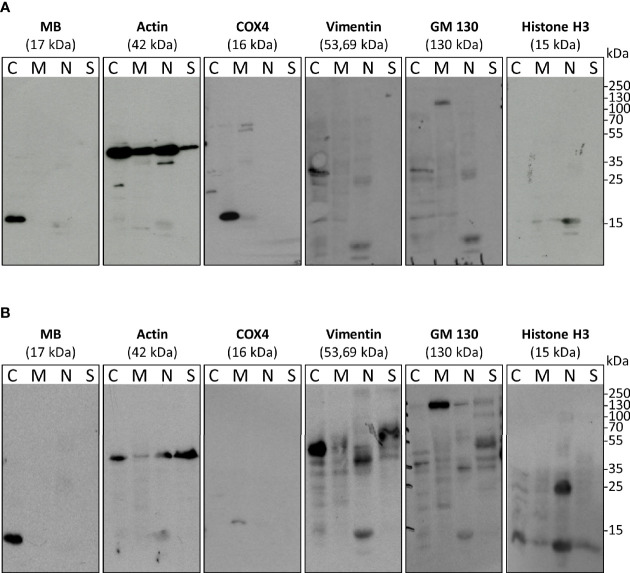
Subcellular fractionation and Western blots with antibodies against myoglobin (MB) and cell compartment-specific marker proteins (histone H3 – nucleus, actin – abundant, Cox4 – mitochondria, Vimentin – cytoplasm and cytoskeleton, GM130 – membrane. MDA-MB-468 cells were cultured under normoxia **(A)** or 1% O_2_ (=hypoxia) **(B)** for 72 h. Fractions include: C, cytoplasm; M, membrane and mitochondria; N, nucleus; S, cytoskeleton.

Although it is known that MB is a cytoplasmic hemoprotein in cardiac myocytes and skeletal muscle fibers, the exact cellular distribution of MB in the more recently analyzed tumor cell lines has not been confirmed. As new studies emerged linking MB to oxidative stress and interactions with the transcription factor hypoxia-inducible factor-1α (13, 31) in tumor cells, we analyzed the cellular distribution of MB in the high-MB breast cancer cell line MDA-MB-468 under both normoxia (NOX, 21% O_2_) and hypoxia (HOX, 1% O_2_). Subcellular fractionation of normoxic or hypoxia-raised MDA-MB-468 cells followed by Western Blot detection of MB and different cell compartment-specific marker proteins revealed that MB was consistently located in the cytoplasm. It was found neither in the nucleus, nor in the mitochondria, membrane or cytoskeleton fractions (**Figure 1**). The same was seen for LNCaP cells, a human prostate adenocarcinoma cell line (**Supplementary Figure 1**). Although MB is thus highly unlikely to directly modulate gene regulation inside the nucleus, the globin might nevertheless be capable of modifying the activation of other transcription factors, which are transiently located in the cytoplasm.

### 3.2 MB Is Important for Cancer Cell Survival

**Figure 2:**
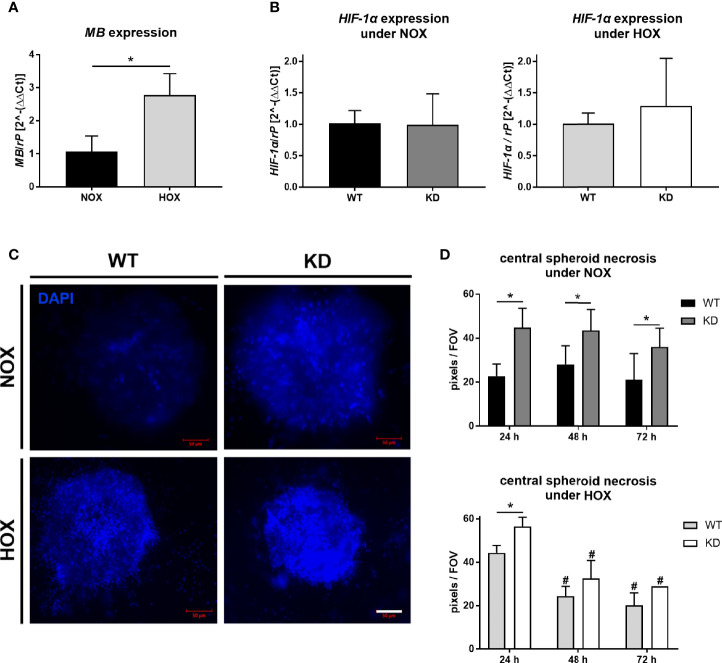
Effect of MB under hypoxia. **(A)** Expression level of *MB* mRNA in MDA-MB-468 monolayer cells after 24 h of normoxic (NOX; 21% O_2_) or hypoxic (HOX, 1% O_2_) incubation. MB expression was significantly increased under HOX conditions. Expression normalized to ribosomal protein S16 (rP); mean +/- sd; n = 3, *p < 0.05.. **(B)** Expression level of HIF-1α in wildtype (WT) or MB-knockdown (KD) MDA-MB-468 monolayer cells after 24 h of normoxia or hypoxia. Knockdown of MB was introduced by transfecting cells with Hs_MB_6 FlexiTube siRNA prior to NOX/HOX incubation, WT cells were transfected with control siRNA. HIF-1α expression remained stable under both NOX and HOX conditions. Expression normalized to ribosomal protein S16 (rP); mean +/- sd; n = 14 in 4 independent experiments (NOX) and 4-5 in 2 independent experiments (HOX). **(C, D)** Central necrosis of WT and KD MDA-MB-468 spheroids was visualized by DAPI intake after 24 h (left), 48 h or 72 h of either normoxia or hypoxia. KD spheroids were transfected with Hs_MB_6 FlexiTube siRNA 5nmol prior to NOX/HOX incubation, WT cells were transfected with control siRNA. MB significantly protected cells from dying especially under NOX conditions, whereas under HOX conditions to a lesser extent. Scale bar = 50 µm. Results are shown as pixels/field of view (FOV), mean +/- sd, n = 4-9 in 4 independent experiments (NOX) and 1 - 6 in 1 experiment (HOX), *p < 0.05 as indicated; ^#^p < 0.05 compared to the 24 h time-point.

As tumor cells in general and MB are both influenced by cellular oxygen levels, we checked the expression of *MB* under NOX compared to HOX conditions in the MDA-MB-468 monolayer cells. *MB* mRNA expression was significantly increased up to three times after 24 h of hypoxic incubation (**Figure 2A**). To further elucidate the effects of MB in hypoxia, we used transient RNA interference to generate MB knockdown (KD) cells. First, we looked for HIF-1α mRNA expression, which remained stable under both normoxia and hypoxia, independent of MB levels (**Figure 2B**). One major characteristic of solid tumors is the formation of a central necrotic, hypoxic core, and an outer proliferating rim zone. We therefore investigated the influence of hypoxia and MB on this central necrosis by generating tumor spheroids, which mimic the tumor architecture *in vitro*. DAPI uptake revealed that the central core region of the spheroids showed a significant increase in dead cells after MB KD compared to WT spheroids, which was stable over 72 h of culturing. Under HOX conditions, this effect was still significantly visible after 24 h of incubation but diminished over 72 h of hypoxia (**Figures 2C, D**). Although after 24 h of hypoxia the central necrosis was initially higher as compared to NOX conditions, prolonged hypoxia led to a significant reduction of the overall central necrosis for both MB WT and KD cells (**Figure 2D**). Thus, MB significantly protected cells from dying especially under normoxic conditions.

### 3.3 MB Protects Cells From ROS

**Figure 3:**
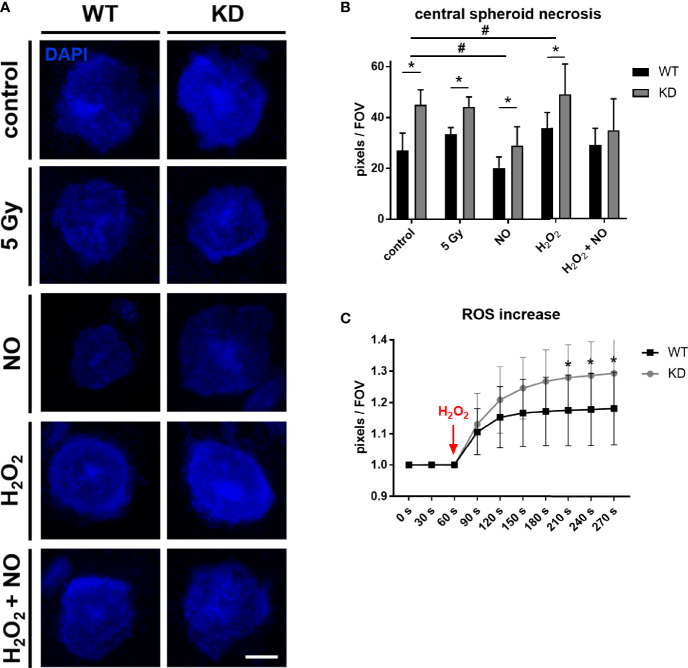
Effect of MB in oxidative stress. **(A, B)** WT and MB KD MDA-MB-468 spheroids were challenged with different substances inducing oxidative stress. 24 h after transfection with Hs_MB_6 FlexiTube siRNA (KD) or control siRNA (WT), cells were either left untreated (control), irradiated with 5 Gy or treated with 500 µM nitric oxide (NO), 100 µM hydrogen peroxide (H_2_O_2_) or a combination of both for 4 h. Central necrosis of spheroids was visualized by DAPI intake. NO treatment led to an overall reduction in central necrotic cells whereas H_2_O_2_ treatment enhanced cellular death. Still, MB protected cells from dying in all tested conditions. Scale bar = 50 µm. Results are shown as pixels/field of view (FOV), mean +/- sd, n = 7-11 in 2 independent experiments, *p < 0.05 between genotypes; ^#^p < 0.05 between treatment conditions. **(C)** 24 h after first transfection, WT and KD MDA-MB-468 monolayer cells were re-transfected with pC1-HyPer3, incubated for another 24 h under normoxia and finally challenged with 100 µM H_2_O_2_ to simulate hypoxic stress leading to an increased ROS formation. ROS levels were measured as changes in fluorescence of the HyPer3 plasmid during live cell imaging over a period of 270 s. Myoglobin knockdown led to significantly higher ROS levels in the cells after treatment with 100 µM H_2_O_2_. Mean +/- sd, n = 12 in 2 independent experiments.

To further mimic tumor microenvironmental stress besides sole hypoxia, we treated the tumor spheroids with different substances inducing oxidative stress. NO is a free radical involved in many physiological and pathophysiological processes. It was also shown to interact with MB (38) and can regulate HIF-1α accumulation and activity (26, 39). H_2_O_2_ is a key metabolite in redox sensing and regulation and leads to the formation of ROS (40). Spheroids were furthermore irradiated with 5 Gy for imitation of irradiation therapy which plays an important role in tumor treatment. Again, we analyzed the central necrosis of WT and KD spheroids. Overall, treatment with NO led to a significant reduction in central necrosis compared to untreated conditions, whereas H_2_O_2_ treatment enhanced the central necrosis. A combination of NO + H_2_O_2_ treatment abrogated these effects. MB significantly protected cells from dying under control conditions as well as under irradiation therapy or treatment with NO or H_2_O_2_ (**Figures 3A, B**). We furthermore measured ROS levels in tumor spheroids after addition of H_2_O_2_ with help of a genetically encoded H_2_O_2_ probe using live cell microscopy. As seen in **Figure 3C**, ROS levels were significantly increased in MB KD spheroids in contrast to WT spheroids. Therefore, MB protected cells from oxidative stress, possibly by its ROS scavenging capabilities.

### 3.4 MB Knockdown Elevates HIF-1α Stabilization After NO Treatment

**Figure 4:**
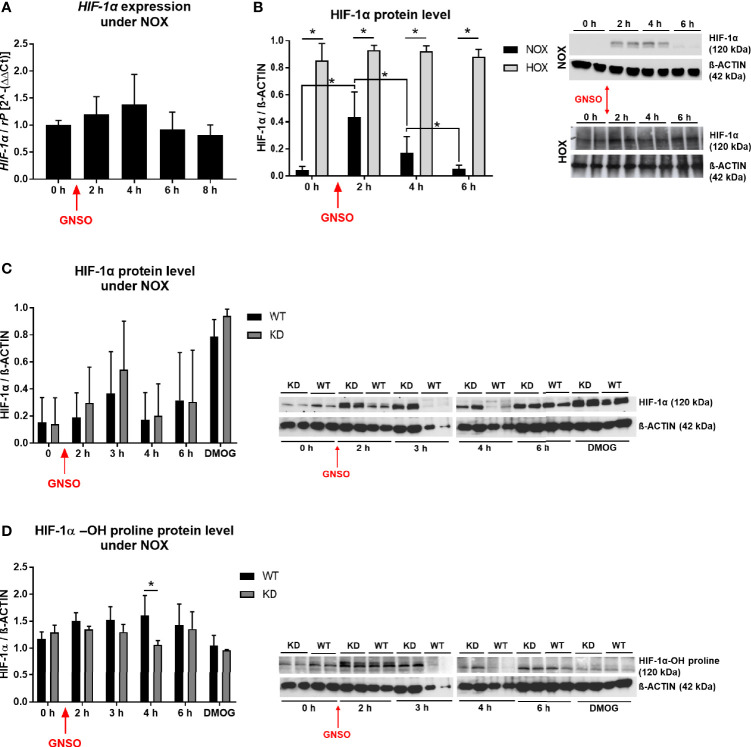
HIF-1α under NO treatment. HIF-1α expression level **(A)** and HIF-1α protein level **(B)** in WT MDA-MB-468 monolayer cells treated with 500 µM GNSO for up to 8 h under normoxia (21% O2) and for protein analysis also hypoxia (1% O2), where cells had already been under HOX for 24 h before addition of GNSO. HIF-1α expression was not altered after GNSO addition. Expression normalized to ribosomal protein S16 (rP); mean +/- sd; n = 4 - 6 in 3 independent experiments. Under normoxia, HIF-1α protein significantly increased 2 h after addition of GNSO while reaching basal levels again after 6 h. Under hypoxia, overall HIF-1α significantly increased compared to normoxic conditions whereas GNSO addition led to no additional changes of HIF-1α level. HIF-1α (120 kDa) normalized to ß-ACTIN (42 kDa) as house-keeping gene; mean +/- sd; n = 12 in 4 independent experiments (NOX) and n = 4 in 2 independent experiments (HOX). **(C, D)** Overall HIF-1α and hydroxylated HIF-1α-OH protein levels of WT and MB KD MDA-MB-468 monolayer cells treated with 500 µM GNSO for up to 6 h under normoxia. 3 h after addition of GNSO, both WT and KD cells showed their highest overall HIF-1α level, with a higher tendency of KD cells compared to WT cells. n = 6-10 in 5 independent experiments (DMOG: n = 2). In contrast, hydroxylated HIF-1α-OH showed an increase only in WT cells whereas in KD cells the level remained constant and significantly lower at 4 h time-point. Addition of dimethyloxaloylglycine (DMOG), a prolyl hydroxylase-inhibitor, was used as a positive control of HIF-1α stabilization. HIF-1α/HIF-1α-OH (120 kDa) normalized to ß-ACTIN (42 kDa) as house-keeping gene; mean +/- sd; n = 4 in 2 independent experiments. *p < 0.05 as indicated.

As we found an association between MB and reduced cell death after NO exposure, and since NO can affect HIF-1α in different ways, we investigated the relationship between NO exposure, MB KD and HIF-1α levels in more detail. In normoxic MDA-MB-468 monolayer WT cells, NO addition did not elicit changes in *HIF-1α* mRNA levels (**Figure 4A**), but a significant stabilization of HIF-1α protein 2 h and 4 h after addition, as shown by Western blot analysis. This transient effect was reduced to basal HIF-1α levels again after 6 h (**Figure 4B**). Cells that had already been exposed to hypoxia for 24 h before treatment with NO kept their high HIF-1α protein level and showed no further enhancement through NO addition (**Figure 4B**). After we saw a clear effect of NO on HIF-1α stabilization under normoxic incubation conditions, we next tested whether knockdown of MB would affect the action of NO. Western blot analysis revealed a transient HIF-1α accumulation for both WT and KD cells after NO treatment, which peaked at 3 h after addition (**Figure 4C**). Thereby, KD cells showed a higher, but not significant, tendency in HIF-1α levels compared to WT cells that was also visible for cells treated with dimethyloxalylglycine (DMOG), a prolyl-hydroxylase-inhibitor that is commonly used as a positive control of HIF-1α stabilization (**Figure 4C**). Next, we addressed the influence of MB KD on the degradation status of HIF-1α. By using the same experimental set-up as mentioned above, we investigated the status of hydroxylated HIF-1α with the help of a specific HIF-1α-OH-proline antibody in Western blot analysis. Whereas hydroxylated HIF-1α protein levels were transiently increased after addition of NO in WT cells, levels of HIF-1α-OH in KD cells remained constant over time and significantly lower compared to WT cells after 4 h of NO incubation (**Figure 4D**). Conclusively, NO treatment led to HIF-1α stabilization that is elevated through a knockdown of MB accompanied by a reduced HIF-1α hydroxylation.

### 3.5 MB Knockdown Leads to the Induction of Hypoxia Signaling Genes After NO Treatment

**Figure 5:**
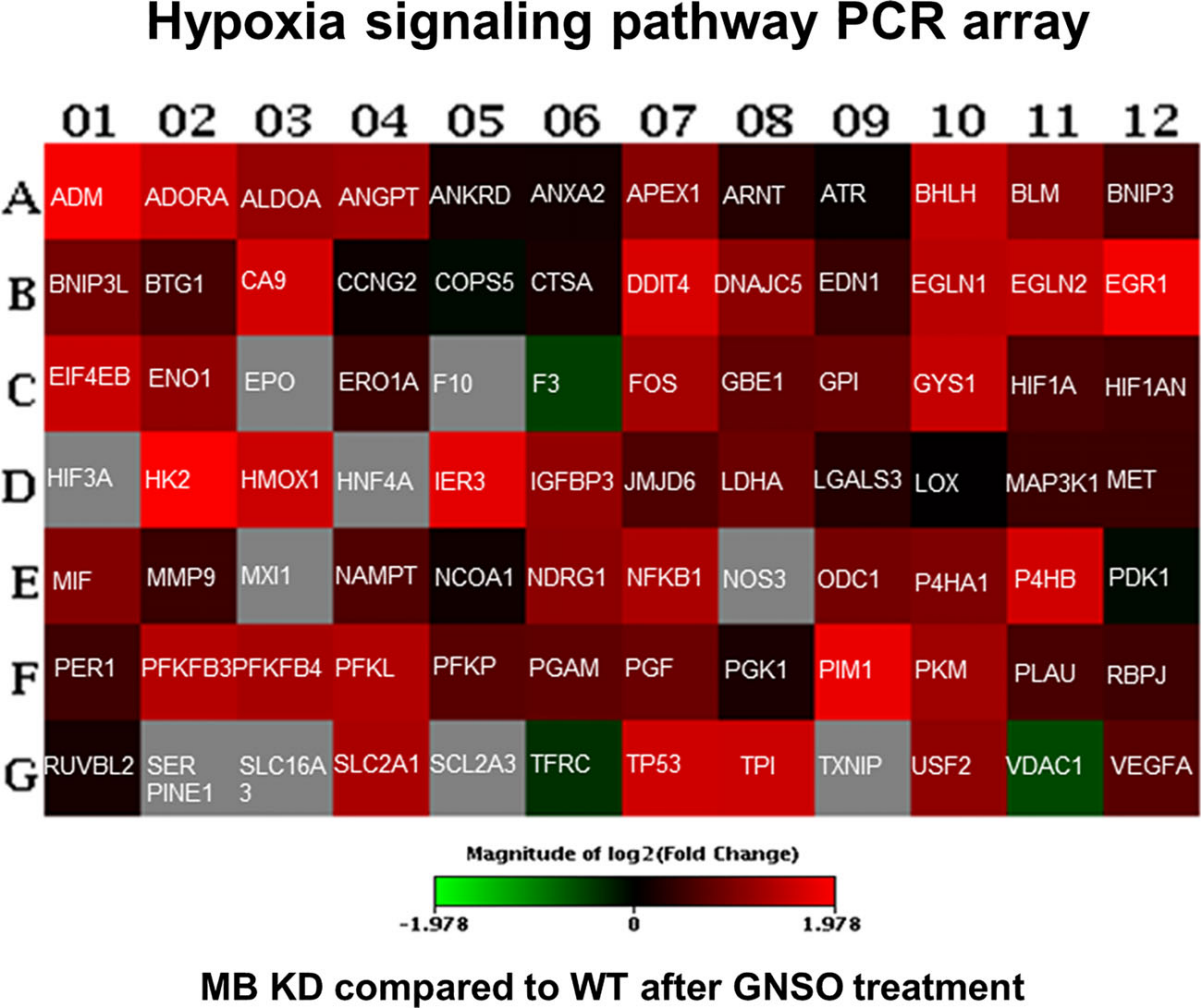
Human hypoxia signaling pathway analysis. WT and KD MDA-MB-468 cells were treated with 500 µM GNSO for 3 h to stimulate HIF-1α accumulation. Changes in expression of 84 genes related to hypoxic signaling were analyzed between KD and WT cells using the RT² Profiler™ PCR Array Human Hypoxia Signaling Pathway (Qiagen). After MB knockdown, most of the genes involved in hypoxic signaling showed an increased expression compared to WT cells. Changes are represented in a heat-map format giving the log 2 fold changes with green = underexpression in KD cells compared to WT cells, red = overexpression in KD cells compared to WT cells and grey = not analyzable. The exact fold change values are depicted in **Supplementary Table 1**.

To analyze, if the elevated HIF-1α stabilization has also effects on hypoxia target genes, we performed a RT² Profiler™ PCR Array for Human Hypoxia Signaling Pathways (Qiagen) for WT and MB KD cells. Cells were treated with NO as stimulator for HIF-1α accumulation for 3 h as this time point showed the highest differences between both conditions. In KD cells, a majority of genes showed an increased expression when compared to WT cells (**Figure 5** and **Supplementary Table 1**). Thus, MB knockdown not only elevated HIF-1α protein stabilization after NO treatment, but consequently also induced the expression of HIF-1 dependent genes involved in hypoxic signaling.

### 3.6 MB Impacts on HIF-1 Target Genes Correlated to Cancer Survival After NO Treatment

**Figure 6:**
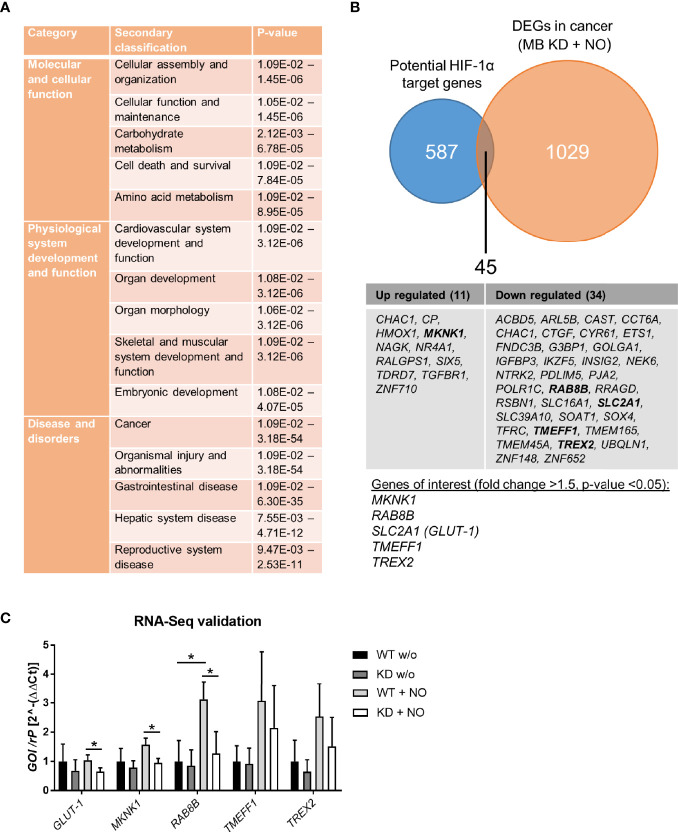
RNA-Seq analysis of MB knockdown cells after treatment with NO. MDA-MB-468 cells were transfected with Hs_MB_6 FlexiTube siRNA (KD) or control siRNA (WT) and subsequently challenged with 500 µM GNSO for 6 h. **(A)** Ingenuity pathway analysis (IPA) showed significantly enriched annotations classified into three categories: molecular and cellular function, physiological system development and function, and disease and disorders. **(B)** 1029 differentially expressed genes (DEGs) of the cancer network were compared to 587 potential HIF-1α target genes revealing 45 genes that were altered by a combination of MB KD and NO treatment. Of these, five genes were identified as genes of interest with a fold change >1.5 and p-value < 0.05. **(C)** Expression levels of the genes of interest (*SLC2A1/GLUT-1, MKNK1, RAB8B, TMEFF1*, and *TREX2*) were validated by qPCR, showing a significant downregulation for *SLC2A1/GLUT-1, MKNK1*, and *RAB8B* under MB KD conditions. As control, WT and MB KD cells were incubated without NO to show NO-mediated effects. Mean +/- SD, n = 4 in 2 independent experiments. *p < 0.05 as indicated.

Since HIF-1 is a transcription factor with several hundred target genes, we used RNA sequencing to identify altered mRNA expression patterns upon treatment with NO in combination with a MB knockdown in contrast to NO-treated WT cells. The differentially expressed genes (DEGs) were analyzed by Ingenuity pathway analysis (IPA) to gain insight into their molecular mechanisms through annotations of diseases, biological functions, and interaction networks. **Figure 6A** shows the significantly enriched annotations classified into three categories: molecular and cellular function, physiological system development and function, and disease and disorders. We further identified the interaction networks and found networks of cell death and survival, cardiovascular system development and function, and cancer that are relevant for breast cancer development. Specifically, we found 1029 DEGs of the cancer network that were altered by the combination of MB KD and NO treatment (**Figure 6B**). In order to identify whether these DEGs of the cancer category were HIF-1 target genes, we checked 587 known and potential HIF-1α target genes (41). Overlap analysis revealed 45 predicted targets that were changed by the combination of MB KD and NO treatment, in which 11 genes were increased and 34 were decreased in expression. Subsequently, five genes were identified as genes of interest with a fold change >1.5 and a p-value <0.05, namely *MKNK1*, *RAB8B*, *SLC2A1/GLUT-1*, *TMEFF1*, and *TREX2* (**Figure 6B**). We validated the effects of MB knockdown in combination with NO treatment on the expression of these genes by qPCR. To see general effects of NO on gene regulation, we furthermore included WT and MB KD cells without NO treatment as controls. NO treatment alone showed a tendency for upregulation of HIF-1 target genes, especially seen for *RAB8B* (**Figure 6C**). In addition, we found that all five genes were down regulated in MB KD cells compared to WT cells upon NO treatment, with *SLC2A1/GLUT-1*, *MKNK1*, and *RAB8B* being significantly affected (**Figure 6C**). Overall, we saw changes of gene expression in fields important for cancer metabolism and outcome that were highly affected by MB knockdown and NO treatment.

## 4 Discussion

Myoglobin is an oxygen-binding protein primarily located in the cytoplasm of striated muscles, namely cardiac myocytes and skeletal muscle fibers. Besides, *MB* expression was also found to a lower extent in different solid tumors and human cancer cell lines from colon, breast, kidney, lung, and prostate (9–12, 42). With its prosthetic heme-group, MB reversibly binds oxygen and functions as O_2_ storage and transporter to the mitochondria of muscle cells, especially under high-energy demand. Myoglobin-facilitated oxygen diffusion furthermore supports oxygen transport from the sarcolemma to the mitochondrial surface (1, 2, 43). MB can furthermore act as an NO dioxygenase or nitrite reductase, depending on the oxygenation status of the iron atom of the heme-group (3–5), or as a scavenger of ROS (6) to reduce oxidative stress in the cells.

The exact role of MB in cancer cells is not fully understood so far, although high expression of MB in tumors is correlated to a better patient prognosis (12). Early findings of Galluzzo et al. in a xenograft mouse model showed reduced hypoxia, minimal HIF-1α levels, and a homogeneously low vessel density in tumors after MB overexpression (44). In cell culture experiments, Kristiansen and colleagues found MB to be hypoxia-inducible and involved in controlling oxidative cell energy metabolism and mitochondrial activity (13). Additional findings showed an inhibition of breast cancer cell proliferation due to an induction of mitochondrial fusion (45), and a positive correlation of MB and HIF-1α signaling and effects on ROS and NO homeostasis in a transcriptomics approach (31).

### 4.1 HIF-1α Abundance Under MB Knockdown and NO Treatment

In our study, we therefore focused on the interplay between MB and HIF-1α and different oxidative-stress inducing agents in the high-MB breast cancer cell line MDA-MB-468. Consistent with other studies (13, 14), MDA-MB-468 cells showed a strong induction of *MB* expression under 1% hypoxia (**Figure 2A**). As the function of MB in carcinoma cells seems to be different from those in muscle cells and because of rising evidence of a functional correlation between MB and transcription factor HIF-1α, we verified the cellular localization of MB in breast carcinoma cells. We found MB to be exclusively expressed in the cytoplasm of MDA-MB-468 cells, independent of their oxygenation status (**Figure 1**), consistent with the current knowledge of MB as a cytoplasmic protein in muscle cells. Therefore, MB unlikely has a direct influence on the activity of the transcription factor itself. A possible interaction needs to take place in the cytoplasm. This fits to the specific regulation of HIF-1 stabilization on a post-translational level, where under normoxic conditions the alpha subunit is targeted for proteasomal degradation by hydroxylation through prolyl-hydroxylases in the cytoplasmic compartment. In line with these findings, *HIF-1α* mRNA expression did not show alterations between WT and MB KD cells under both NOX and HOX conditions (**Figure 2B**).

NO is a molecule important for the regulation of many processes in inflammation and oxidative responses but is also known for its diverse effects in cancer. Depending on cancer type, concentration, and timing, NO can have either anti-tumor or carcinogenic effects. For breast cancer, NO seems to have a rather cancer-promoting role and the NO pathway is also interacting with steroid hormones in different ways (46). HIF-1α stabilization is not only achieved through hypoxic stimuli, but also through NO signaling as shown in different studies and for different cell lines (47–50). It is therefore imaginable that tumor-associated NO modulates HIF-1α abundance in tumors. Moreover, opposite effects of HIF-1α stabilization were visible under hypoxia, where NO treatment led to a decrease in HIF-1α activity. Berchner-Pfannschmidt and colleagues proposed a bimodal system, where in the early phase, NO inhibits PHD activity that leads to HIF-1α accumulation, whereas in the late phase, increased PHD levels reduce HIF-1α through a negative feedback loop (39). When we studied HIF-1α stabilization after NO exposure in the MDA-MB-468 cells, we found an induction of HIF-1α protein levels which transiently peaked around 2 h to 3 h after GNSO addition (**Figure 4B**) and returned to basal levels after 6 h. *HIF-1α* mRNA levels remained constant over time (**Figure 4A**). In addition, when we cultured the cells under hypoxia, NO treatment led to no further stabilization of HIF-1α as HIF-1α levels were already very high, but also led to no degradation as observed for U2OS osteosarcoma cells (**Figure 4B**) (39). Of note, though we applied a similar experimental protocol we used a different cancer cell line that may react differently or show different kinetics. Because oxygenated MB can scavenge NO, we compared HIF-1α levels between WT and MB KD cells after NO treatment. We found that the MB knockdown elevates the HIF-1α stabilization after NO treatment (**Figure 4C**), accompanied by decreased hydroxylation of HIF-1α (**Figure 4D**) which fits to the mode of action of NO as an inhibitor of prolyl hydroxylases. necrosis is an important hallmark of aggressiveUnder physiological conditions, MB therefore seems to metabolize NO that is then to some extent missing for inhibition of PHDs and an enhanced HIF-1α stabilization.

### 4.2 Expression of HIF-1α Target Genes Under MB Knockdown and NO Treatment

Not only abundance of HIF-1α was altered through exposure to NO and knockdown of MB but also target gene expression (**Figure 5**). These findings show that MB is indeed capable of NO scavenging in breast carcinoma cells and can thereby influence HIF-1 activity. We therefore took a deeper look into altered gene expression levels *via* RNA-Seq analysis and searched for correlations between genes of the cancer category and potential HIF-1 target genes. We found that MB affects HIF-1 target genes correlated to cancer survival with up regulated but also down regulated examples (**Figures 6A, B**). Surprisingly, when verified by qPCR, we found a down regulation of *GLUT-1*, *MKNK-1*, and *RAB8B* after MB KD (**Figure 6C**). These genes are often up regulated in cancer and correlate with a poor clinical outcome (32–34). *GLUT-1* is a very prominent HIF-1 target gene that is normally up regulated through HIF-1. As we saw an extended HIF-1 stabilization after MB KD and NO treatment, we would also have expected mostly up regulations of HIF-1 target genes, as seen in the Qiagen array (**Figure 5**). However, this was not corroborated by RNA-Seq (**Figure 6C**). This discrepancy needs to be further addressed. In addition, when Bicker et al. analyzed gene expression of MB knockdown *vs*. wildtype MDA-MB-468 cells at 1% hypoxia in a transcriptomics approach, they found increased cellular activity of HIF-1 signaling in WT cells (31). At first sight, this seems to contradict to our findings, where we showed enhanced HIF-1α levels in the MB KD, but not wildtype cells. On the other hand, Bicker and colleagues performed their experiments under hypoxic conditions without external NO addition, whereas we looked for HIF-1α stabilization after NO treatment under normoxic conditions. Indeed, our findings match the proposed working model of MB presence and HIF-1α activity of Bicker et al. when we take into account that MB can function as a nitrite reductase, or NO dioxygenase based on the redox status. Under hypoxic conditions, deoxygenated MB can function as nitrite reductase leading to production of NO and the above-discussed indirect stabilization of HIF-1α, as seen in the Bicker et al. model, whereas in our case, oxygenated MB mainly serves as an NO dioxygenase, which scavenges NO that is therefore missing for HIF-1α stabilization in wildtype cells. When looking for crosstalk between MB and HIF-1α especially in the tumor environment, one has thus to pay close attention to the specific oxygenation status and its influence on NO metabolism.

### 4.3 Interplay Between MB Knockdown, Oxidative Stress and Cancer Cell Survival

Tumor necrosis is an important hallmark of aggressive cancers and associated with poorer patient prognosis in different types of cancer (51–54). By using tumor spheroids, which mimic the 3D architecture of a tumor *in vitro*, we looked for the abundance of the central necrotic core with or without *MB* expression under both normoxia and 1% hypoxia. We found that MB is important for cell survival as MB knockdown led to a significant increase in the central necrotic area of the spheroids (**Figure 2D**). This was especially true for normoxic conditions, but also visible under hypoxia. The central necrotic core and surrounding rim zone of a tumor are known for their lack of oxygen. As a knockdown of MB is increasing the size of the central necrotic core especially under normoxic conditions, one could conclude that in MDA-MB-468 breast cancer cells MB also uses its O_2_ storage and transport capacities for survival of cancer cells. This would fit to the xenograft model of Galluzzo et al. who found *MB* expression correlated with better tumor oxygenation (44), and to the general link between high MB levels, low hypoxia and good therapeutic response in cancer patients. Additionally, Yang et al. showed that myoglobin-containing particles increased the intracellular oxygen levels of A549 cells, thereby sensitizing them to radiation. Their findings suggested an effective approach to enhance efficiency of radiotherapy by usage of genetically modified O_2_-bound myoglobin particles, and also showed an O_2_ transport function of MB in tumor cells (55). When we treated the spheroids with irradiation, NO or H_2_O_2,_ the knockdown of MB led to greater central necrosis compared to WT cells (**Figure 4B**). Overall necrosis was thereby diminished upon NO treatment but enhanced through H_2_O_2_.

In general, effects of NO on cell death depend on cell type, cellular redox status and NO dosage (56). In our tumor cell model, NO had a beneficial effect on cell survival. H_2_O_2_ is frequently used in cell culture to mimic oxidative stress. Next to other ROS, like superoxide, H_2_O_2_ is prone to induce cell death, for example through TNF- α signaling, in different cells (57–59), although its role in cancer development is ambivalent (60). In the MDA-MB-468 tumor spheroids, H_2_O_2_ reduced cell survival. By using a H_2_O_2_ sensor plasmid, we measured ROS levels in the spheroids after addition of H_2_O_2_ and found a significant difference between WT and MB KD spheroids, with enhanced ROS levels after MB KD (**Figure 4C**). MB is therefore not only able to scavenge NO but also H_2_O_2_ in breast cancer cells, leading to beneficial effects on cell survival under oxidative stress. Nevertheless, high-MB breast tumors have a better prognostic outcome, which may be related to better responsiveness to chemo- or radiotherapy.

## 5 Conclusion

Our study is limited because we studied only one breast cancer cell line. Certainly, more studies are necessary to better understand the role of NB in breast cancer disease. In that respect, it is interesting that in patients with lung adenocarcinoma, high MB levels were linked to a poorer patient prognosis (10), indicating cancer-specific differences of MB function. Furthermore, HIF-1α is considered to have either pro- or anti-apoptotic effects depending on for example the severity of hypoxia. Besides activation of target genes inducing apoptosis itself, HIF-1α shows contrary interactions with the tumor suppressor protein p53 that is also linked to apoptosis (61, 62).In their transcriptome study, Bicker and colleagues found GO-terms associated with negative as well as positive p53-mediated regulation of apoptosis in the MDA-MB-468 cells, linking MB to this mechanism (31). Off note, MDA-MB-468 cells exhibit a gain-of-function mutation of p53 with R273H that also be taken into account (63). Since our study contained experiments on necrosis and cell survival, it would be beneficial to address apoptosis further and the interplay of MB, HIF-1α and p53 in this process in the MDA-MB-468 breast cancer cells.

Taken together, our study is in agreement the work of Flonta and colleagues who proposed several possible mechanisms of MB function in tumor cells, that is as regulator of oxidative stress, O_2_ transporter to overcome hypoxia, or by regulation of HIF-1α (42). Indeed, our study found evidence for all three assumptions in breast cancer cells, illustrating the diverse modes-of-action of MB in cancer metabolism and progression. Although precise mechanisms need to be further elucidated, our data indicate that myoglobin plays an important role in survival of breast cancer cells, possibly due to its ROS and NO scavenging properties and might be a valuable target for cancer therapy.

## Data Availability Statement

The datasets presented in this study can be found in online repositories. The names of the repository/repositories and accession number(s) can be found below: https://www.ebi.ac.uk/metagenomics/, PRJEB45683.

## Author Contributions

AH performed and analyzed the experiments, unless otherwise stated. TQ analyzed part of the data and wrote the manuscript. TN and ABi performed cellular localization experiments. ABi performed RNA-Seq experiments and analysis. ABe conducted the ROS measurements. TH analyzed part of the data and supported the project. JF supervised and supported the project. TS conceived the project and designed the experiments. All authors discussed the results and commented on the manuscript. All authors contributed to the article and approved the submitted version.

## Funding

The study was supported by the Deutsche Forschungs gemeinschaft DFG Ha2103/10-1 (ABi and TH).

## Conflict of Interest

The authors declare that the research was conducted in the absence of any commercial or financial relationships that could be construed as a potential conflict of interest.

## Publisher’s Note

All claims expressed in this article are solely those of the authors and do not necessarily represent those of their affiliated organizations, or those of the publisher, the editors and the reviewers. Any product that may be evaluated in this article, or claim that may be made by its manufacturer, is not guaranteed or endorsed by the publisher.

## Abbreviations

BSA, bovine serum albumin; DEG, differentially expressed genes; DMOG, dimethyloxaloylglycine; HIF, hypoxia-inducible factor; HOX, hypoxia; IPA, Ingenuity pathway analysis; KD, knockdown; MB, myoglobin; NO, nitric oxide; NOX, normoxia; PHD, prolyl-hydroxylase; qPCR, quantitative real-time PCR; ROS, reactive oxygen species; WT, wildtype.
