# Supplementary material for: Myoglobin Protects Breast Cancer Cells Due to Its ROS and NO Scavenging Properties
Source: Front Endocrinol (Lausanne). 2021 Oct 4;12:732190. doi: 10.3389/fendo.2021.732190 (PMC8521001; doi:10.3389/fendo.2021.732190)
Supplement: Supplementary file 1 [file DataSheet_1.docx]

**
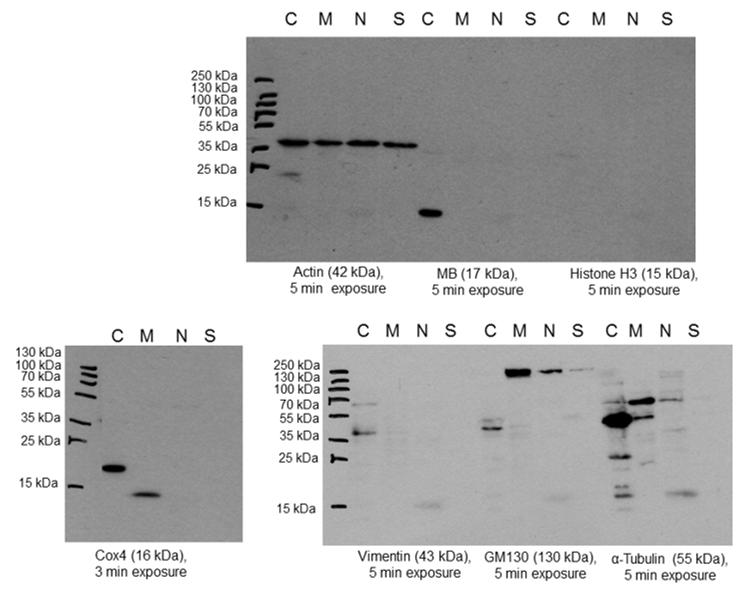
**

**Supplementary** **Figure 1.** Subcellular fractionation and Western blots with antibodies against myoglobin (MB) and cell compartment-specific marker proteins (histone H3 – nucleus, actin – abundant, Cox4 – mitochondria, α-Tubulin, Vimentin – cytoplasm and cytoskeleton, GM130 – membrane. LNCaP cells were cultured under 1% O_2_ (=hypoxia) for 72 h. Fractions include: C = cytoplasm; M = membrane and mitochondria; N = nucleus; S = cytoskeleton.

**Supplementary Table 1.** Gene layout with exact fold change values of the human hypoxia signaling pathway analysis.

| Layout | 1 | 2 | 3 | 4 | 5 | 6 | 7 | 8 | 9 | 10 | 11 | 12 |
| --- | --- | --- | --- | --- | --- | --- | --- | --- | --- | --- | --- | --- |
| A | ADM 3.78 | ADORA2B 3.01 | ALDOA 2.25 | ANGPTL4 2.34 | ANKRD37 1.12 | ANXA2 1.10 | APEX1 2.08 | ARNT 1.21 | ATR 1.04 | BHLHE40 2.86 | BLM 2.05 | BNIP3 1.43 |
| B | BNIP3L 1.89 | BTG1 1.46 | CA9 3.11 | CCNG2 1.07 | COPS5 -1.06 | CTSA 1.14 | DDIT4 3.22 | DNAJC5 2.14 | EDN1 1.35 | EGLN1 2.73 | EGLN2 2.84 | EGR1 3.70 |
| C | EIF4EBP1 2.96 | ENO1 2.19 | EPO 1.01 | ERO1A 1.37 | F10 1.01 | F3 -1.40 | FOS 2.42 | GBE1 1.67 | GPI 1.75 | GYS1 2.90 | HIF1A 1.49 | HIF1AN 1.41 |
| D | HIF3A 1.01 | HK2 3.94 | HMOX1 3.01 | HNF4A 1.01 | IER3 3.41 | IGFBP3 2.22 | JMJD6 1.52 | LDHA 1.77 | LGALS3 1.21 | LOX 1.01 | MAP3K1 1.36 | MET 1.36 |
| E | MIF 2.04 | MMP9 1.36 | MXI1 1.01 | NAMPT 1.55 | NCOA1 1.09 | NDRG1 2.13 | NFKB1 2.51 | NOS3 1.01 | ODC1 1.86 | P4HA1 1.97 | P4HB 3.09 | PDK1 -1.05 |
| F | PER1 1.40 | PFKFB3 2.67 | PFKFB4 2.48 | PFKL 2.60 | PFKP 1.60 | PGAM1 1.66 | PGF 1.76 | PGK1 1.15 | PIM1 3.55 | PKM 2.36 | PLAU 1.47 | RBPJ 1.40 |
| G | RUVBL2 1.13 | SERPINE1 1.01 | SLC16A3 1.01 | SLC2A1 2.53 | SLC2A3 1.01 | TFRC -1.29 | TP53 3.07 | TPI1 2.98 | TXNIP 1.01 | USF2 2.17 | VDAC1 -1.48 | VEGFA 1.60 |
